# High‐intensity exercise in hypoxia improves endothelial function via increased nitric oxide bioavailability in C57BL/6 mice

**DOI:** 10.1111/apha.13700

**Published:** 2021-06-19

**Authors:** Jessica Lavier, Manon Beaumann, Steeve Menétrey, Karima Bouzourène, Nathalie Rosenblatt‐Velin, Vincent Pialoux, Lucia Mazzolai, Anne‐Christine Peyter, Maxime Pellegrin, Grégoire P. Millet

**Affiliations:** ^1^ Division of Angiology, Heart and Vessel Department Lausanne University Hospital (CHUV) Lausanne Switzerland; ^2^ Institute of Sport Sciences University of Lausanne Lausanne Switzerland; ^3^ Neonatal Research Laboratory Clinic of Neonatology Department Woman‐Mother‐Child Lausanne University Hospital (CHUV) Lausanne Switzerland; ^4^ Inter‐University Laboratory of Human Movement Biology EA7424 University of Lyon Villeurbanne France

**Keywords:** endothelial function, exercise training, high‐intensity, hypoxia, nitric oxide bioavailability

## Abstract

**Aim:**

The optimal exercise intensity to improve endothelial function remains unclear, as well as whether the addition of hypoxia could potentiate this function. Therefore, the aim of this study was to compare the effects of different exercise intensities in normoxia and hypoxia on vascular reactivity and nitric oxide (NO) bioavailability in mice.

**Methods:**

C57BL/6 mice underwent treadmill running three times per week, for 4 weeks at either low, maximal or supramaximal intensity in normoxia or hypoxia (inspire oxygen fraction = 0.13). Vascular reactivity and expression of genes and proteins involved in NO production/bioavailability were assessed in aorta using isolated vessel tension experiments, RT‐qPCR and western blot, respectively. Circulating NO metabolites and pro‐/antioxidant markers were measured.

**Results:**

Hypoxic exercise improved both acetylcholine‐induced vasorelaxation and phenylephrine‐induced vasoconstriction compared to normoxic exercise, independently of intensity. In hypoxia, a higher acetylcholine‐induced vasorelaxation was observed with high intensities (supramaximal and maximal) compared to low intensity. Exercise protocols modulated endothelial nitric oxide synthase (eNOS) and α1‐adrenergic receptor (α_1_‐AR) mRNA level, but not superoxide dismutase 3 (SOD3) and p47phox. No significant differences were observed for protein expression of α_1_‐AR, total eNOS, phosphorylated eNOS, SOD isoforms and p47phox. However, plasma SOD and catalase activities were significantly increased in hypoxic supramaximal compared to hypoxic low intensity, while concentration of nitrotyrosine significantly decreased. The latter was also observed in hypoxic maximal and supramaximal compared to the same intensities in normoxia.

**Conclusion:**

Hypoxic high‐intensity exercise increases NO bioavailability and improves vascular function, opening promising clinical perspectives for cardiovascular disease prevention.

## 1 INTRODUCTION

Endothelial function lies in the ability of vessels to contract and relax under thorough control of endothelial cells and is directly linked to vascular health. Since the monolayer of endothelial cells is located between the blood flow and vessel wall components, it is a key regulator of different signaling pathways affecting vascular function and structure.^1^ The most notorious one is the nitric oxide (NO) pathway. NO is synthesized in endothelial cells through the mobilization/activation of endothelial nitric oxide synthase (eNOS).^1^ Impaired eNOS activity and/or reduction of NO bioavailability leads to endothelial dysfunction, an independent risk factor for cardiovascular diseases.^2^ Thus, ameliorating endothelial function or preventing endothelial dysfunction through targeting eNOS and NO bioavailability is of clinical interest for the prevention of cardiovascular disorders.

Exercise training is a highly efficient nonpharmacological approach for maintaining cardiovascular health as well as for the primary and secondary prevention of cardiovascular diseases,^3^ partly via its beneficial effect on endothelial function. There is extensive evidence showing that exercise training improved endothelial function in patients with cardiovascular risk factors or established cardiovascular diseases.^4^, ^5^, ^6^, ^7^ Such beneficial effects have also been reported in animal models.^8^, ^9^, ^10^ Although the mechanisms behind the benefits of exercise training on endothelial function are not fully understood, it has been advocated that exercise training acts on NO bioavailability through shear stress‐induced stimulation of endothelial cells, promoting the activation of eNOS. Increased NO bioavailability induced by exercise training can also be mediated by a reduction of oxidative stress and reactive oxygen species (ROS) production, through decreasing pro‐oxidant agents such as NADPH oxidase subunits and/or increasing antioxidant defenses such as superoxide dismutase (SOD) isoforms and catalase.^10^, ^11^

So far, the optimal characteristics of exercise training needed to improve endothelial function remain unclear. However, vascular reactivity seems to be sensitive to exercise training intensity in humans, with high‐intensity interval‐training improving vascular reactivity more efficiently than lower intensity protocols.^12^, ^13^, ^14^ Few studies in rodents have also reported higher aortic endothelium‐dependent vasorelaxation following either high‐intensity interval training or high‐intensity endurance training compared to moderate intensity endurance training, which was associated with increased eNOS protein expression.^15^, ^16^ On the other hand, other animal studies reported no benefits with high intensity continuous exercise training compared to moderate continuous exercise training, or with high intensity interval training compared to low intensity interval training.^17^, ^18^ Some studies even described a deleterious effect of high intensity continuous training compared to low and moderate intensity continuous training.^19^, ^20^ Thus, there is still controversy regarding the potential beneficial effect of high‐intensity exercise training on endothelial function.

Hypoxia is a state that induces an imbalance between tissue oxygen (O_2_) delivery and demand. Current evidence show that combining exercise training with hypoxia potentiates the vascular adaptations observed with the same level of exercise training in normoxia, including those related to the dilator function.^21^ In fact, hypoxic training produces a “compensatory” vasodilatation and an augmented blood flow aiming at preserving tissue O_2_ delivery, and ensuring it is matched to demand.^22^, ^23^ These hypoxic exercise training ‐induced compensatory vasodilatation and vascular responses have been shown to be mainly mediated by NO.^22^, ^23^

To date, however, there has been a lack of research on the effect of combining high‐intensity exercise training and hypoxia on endothelial function. More specifically, to our knowledge, no study has yet investigated the effects of supramaximal intensity training on endothelial function, in normoxia or in combination with hypoxia. In addition, mechanisms regulating the NO pathways responsible for the effect of hypoxic exercise training on vascular function remain incompletely understood.

Therefore, this study aimed at comparing the effects of different exercise training intensities (low, maximal and supramaximal) in normoxia and in hypoxia on vascular reactivity in mice. We hypothesized that the vascular adaptations would depend on the combination of both exercise training intensity and O_2_ availability, and therefore that (a) vascular reactivity (ie, vasoconstriction and vasodilation) would improve to a greater extent as exercise training intensity increases either in normoxia or hypoxia; and (b) hypoxic exercise training would improve vascular reactivity to a greater extent than the same intensity in normoxia.

## 2 RESULTS

Mice were submitted to a total of 12 training sessions and stayed in hypoxia for approximately 1 h each session. As expected, there was a significant main effect of hypoxia independently of exercise training intensity in mRNA expression of HIF‐1α (*P* < .05; Figure S1).

### 2.1 Body weight gain and mean arterial blood pressure

Body weight (BW) increased significantly in each group of mice between the beginning and end of the study (*P* < .05, data not shown). BW gain was higher in LowH compared to LowN and in MaxH compared to MaxN (*P* < .001 and *P* < .01, respectively; Table S2). No significant differences in BW gain were observed between SupraH and SupraN.

Mean arterial blood pressure did not significantly change between the beginning and end of the study in any of the groups (Table S2).

### 2.2 Vascular reactivity tension studies

#### 2.2.1 Vascular relaxation responses

**FIGURE 1:**
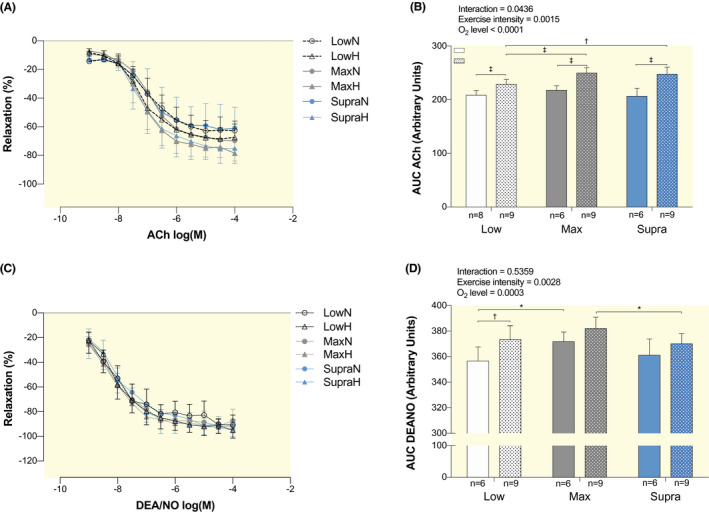
Effect of hypoxic exercise training at different intensities on endothelium‐dependent and endothelium‐independent vasorelaxation. Dose‐response curves to acetylcholine (ACh, A) and to DEA/NO (C) of isolated abdominal aorta pre‐constricted with phenylephrine in LowN, LowH, MaxN, MaxH, SupraN and SupraH mice. Bar graphs show the area under the curve of the vascular responses (B, D), calculated from the relaxation curves shown in A and C. Data are expressed as mean ± SD (n = 6 to 9 mice per group) of the percent of change in tension induced by the vasodilator. Two‐way ANOVA with Sidak post hoc test: *P < .05; ^†^ *P* < .01; ^‡^ *P* < .001. Groups: low intensity training group in normoxia (LowN), low intensity training group in hypoxia (LowH), maximal intensity training group in normoxia (MaxN), maximal intensity training group in hypoxia (MaxH), supramaximal intensity training group in normoxia (SupraN) and supramaximal intensity training group in hypoxia (SupraH)

As shown in Figure 1A,B, the endothelium‐dependent vasorelaxation to ACh was significantly improved in LowH compared to LowN (+10.0%, *P* < .001), as well as in MaxH compared to MaxN (+14.8%, *P* < .0001) and SupraH compared to SupraN (+20.0%, *P* < .0001). ACh‐induced relaxation was 9.2% greater in MaxH and 8.2% greater in SupraH than in LowH (*P* < .001 and *P* < .01, respectively; Figure 1A,B). There was no significant difference in ACh‐induced relaxation between LowN, MaxN, and SupraN (Figure 1A,B).

Endothelium‐independent relaxation to the NO donor DEA/NO was significantly increased in LowH compared to LowN (+4.7%, *P* < .01; Figure 1C,D). It was also significantly greater in MaxN compared to LowN (+4.3%, *P* < .05) and in MaxH compared to SupraH (+3.2%, *P* < .05) (Figure 1C,D).

#### 2.2.2 Vascular constriction responses

**FIGURE 2:**
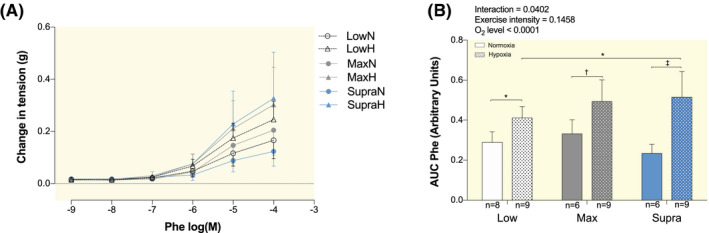
Effect of hypoxic exercise training at different intensities on vasoconstriction. Dose‐response curves to α_1_‐AR agonist Phenylephrine (Phe, A) of isolated aorta of LowN, LowH, MaxN, MaxH, SupraN and SupraH mice. Bar graph shows the area under the curve of the vascular responses (B), calculated from the contraction curves shown in A. Data are expressed as mean ± SD (n = 6 to 9 mice per group). Two‐way ANOVA with Sidak post hoc test: *P < .05; ^†^ *P* < .01; ^‡^ *P* < .001. Groups: low intensity training group in normoxia (LowN), low intensity training group in hypoxia (LowH), maximal intensity training group in normoxia (MaxN), maximal intensity training group in hypoxia (MaxH), supramaximal intensity training group in normoxia (SupraN) and supramaximal intensity training group in hypoxia (SupraH)

The vascular constriction to Phe was significantly improved in LowH, MaxH and SupraH compared to LowN, MaxN and SupraN, respectively (+42.1%, *P* < .05; +48.3%, *P* < .01 and +119.9%, *P* < .0001; Figure 2A,B). Vasoconstriction was greater in SupraH compared to LowH (+25.3%, *P* < .05; Figure 2A,B).

### 2.3 Aortic mRNA and protein expression of markers involved in NO production

**FIGURE 3:**
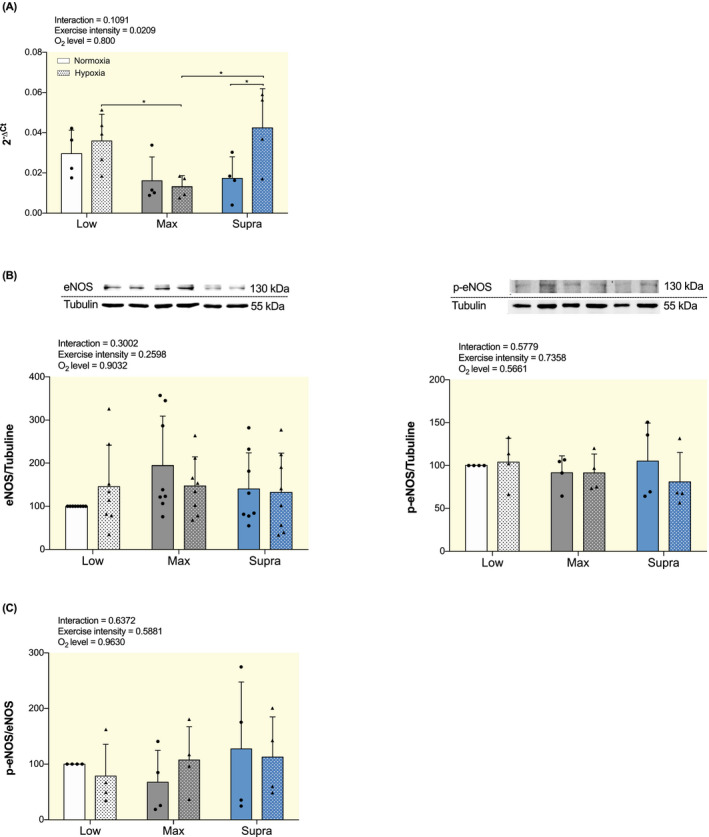
Effect of hypoxic training at different intensities on mRNA and protein expression of markers involved in NO production in aorta. A, Gene expression analysis of eNOS. Results are expressed as 2^−ΔCt^ using 36B4 as housekeeping gene. B, Western blot analysis of eNOS and p‐eNOS protein expression. Protein expressions were normalized to tubulin content in each sample and expressed as the percentage of the protein expression values obtained in the LowN group. Top panel: representative WB images; bottom panel: quantitative analysis. C, Ratio of p‐eNOS to eNOS. Data are presented as mean ± SD (n = 4 to 8 mice per group). Two‐way ANOVA with Sidak post hoc test: *P < .05. Groups: low intensity training group in normoxia (LowN), low intensity training group in hypoxia (LowH), maximal intensity training group in normoxia (MaxN), maximal intensity training group in hypoxia (MaxH), supramaximal intensity training group in normoxia (SupraN), and supramaximal intensity training group in hypoxia (SupraH)

mRNA level of eNOS was higher in SupraH compared to SupraN (*P* < .05), while no significant differences were observed in LowH and MaxH, compared to their respective normoxic groups (Figure 3A). mRNA level of eNOS was significantly higher in LowH and SupraH compared to MaxH (*P* < .05; Figure 3A). No significant difference was observed between LowN, MaxN, and SupraN (Figure 3A). Neither protein expressions of eNOS nor phospho‐eNOS (p‐eNOS) showed significant differences between the six training groups (Figure 3B). The ratio of p‐eNOS to eNOS expression revealed no significant difference between any of the groups either (Figure 3C).

### 2.4 Aortic mRNA and protein expression of markers involved in NO bioavailability

**FIGURE 4:**
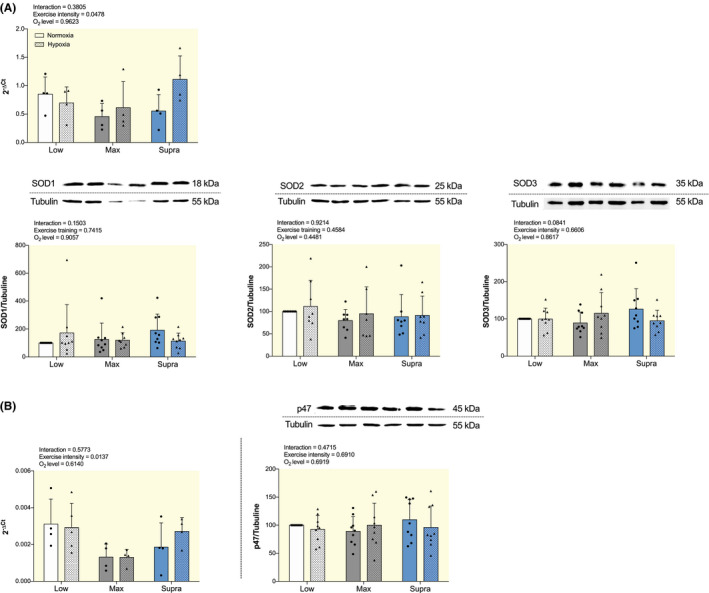
Effect of hypoxic training at different intensities on mRNA and protein expression of markers involved in NO bioavailability in aorta. A, Gene expression analysis of antioxidant SOD3 (top) and Western blot analysis of SOD1, SOD2 and SOD3 protein expression (low). B, Gene expression analysis (left) and Western blot analysis (right) of pro‐oxidant p47phox. For gene expression analysis, results are expressed as 2^−ΔCt^ using 36B4 as housekeeping gene. For western blot analysis, protein expressions were normalized to tubulin content in each sample and expressed as the percentage of the protein expression values obtained in the LowN group. Top panel: representative WB images, and bottom panel: quantitative analysis. Data are presented as mean ± SD (n = 4 to 9 mice per group). Two‐way ANOVA with Sidak post hoc test. Groups: low intensity training group in normoxia (LowN), low intensity training group in hypoxia (LowH), maximal intensity training group in normoxia (MaxN), maximal intensity training group in hypoxia (MaxH), supramaximal intensity training group in normoxia (SupraN), and supramaximal intensity training group in hypoxia (SupraH)

As shown in Figure 4A, there were no significant differences in mRNA level of antioxidant superoxide dismutase 3 (SOD3) as well as in the protein expression of antioxidant SOD1, SOD2 and SOD3 among any of the groups. Neither the mRNA level nor the protein expression of pro‐oxidant p47phox significantly differed between groups (Figure 4B).

### 2.5 Aortic mRNA expression of soluble guanylate cyclase subunit alpha 1 and enzymes involved in hydrogen sulfide production

As shown in Figure S2, there were no significant differences in mRNA level of soluble guanylate cyclase subunit alpha 1 (sGCα1) among any of the groups. Neither mRNA expression of CBS, CSE nor 3MST were significantly different between groups (Figure S3).

### 2.6 Aortic mRNA and protein expression of vasoconstriction marker α_1_‐adrenergic receptor

**FIGURE 5:**
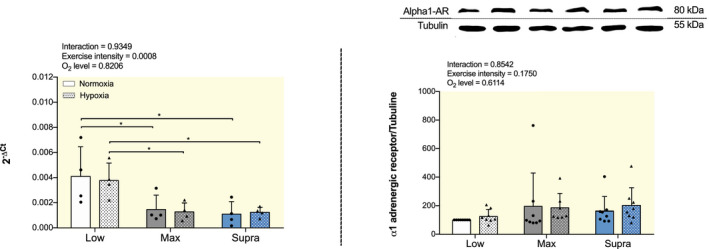
Effect of hypoxic training at different intensities on mRNA and protein expression of vasoconstriction marker α_1_‐AR in aorta. Gene expression analysis (left) and western blot analysis (right) of α_1_‐AR. For gene expression analysis, results are expressed as 2^−ΔCt^ using 36B4 as housekeeping gene. For western blot analysis, protein expressions were normalized to tubulin content in each sample and expressed as the percentage of the protein expression values obtained in the LowN group. Top panel: representative WB images, and bottom panel: quantitative analysis. Data are presented as mean ± SD (n = 4 to 8 mice per group). Two‐way ANOVA with Sidak post hoc test: *P < .05. Groups: low intensity training group in normoxia (LowN), low intensity training group in hypoxia (LowH), maximal intensity training group in normoxia (MaxN), maximal intensity training group in hypoxia (MaxH), supramaximal intensity training group in normoxia (SupraN) and supramaximal intensity training group in hypoxia (SupraH)

The mRNA expression of α1‐adrenergic receptor (α_1_‐AR) was significantly lower in MaxN and SupraN compared to LowN (*P* < .05; Figure 5, left). It was also lower in MaxH and SupraH compared to LowH (*P* < .05; Figure 5, left). There was no significant difference in the expression of α_1_‐AR between normoxic and hypoxic groups for the same exercise training intensity (Figure 5, left). Protein expression of α_1_‐AR remained unchanged between the groups (Figure 5, right).

### 2.7 Circulating NO metabolites, pro‐ and antioxidants markers

**FIGURE 6:**
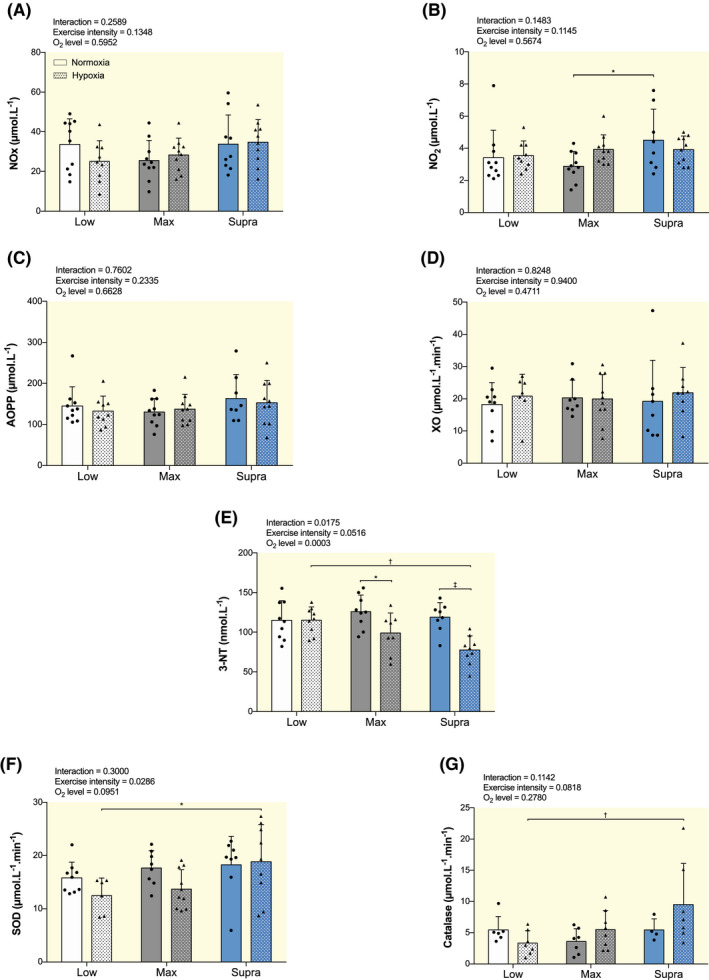
Effect of hypoxic training at different intensities on circulating NO metabolites, oxidative stress markers and antioxidant markers. Plasma concentrations of NO metabolism end‐product nitrite and nitrate (NOx, A), nitrite alone (NO_2_, B), advanced oxidation protein products (AOPP; C), xanthine oxidase (XO; D) and nitrotyrosine (3‐NT, E), and plasma superoxide dismutase (SOD; F) and catalase (G) activities in low intensity training group in normoxia (LowN), low intensity training group in hypoxia (LowH), maximal intensity training group in normoxia (MaxN), maximal intensity training group in hypoxia (MaxH), supramaximal intensity training group in normoxia (SupraN) and supramaximal intensity training group in hypoxia (SupraH). Data are presented as mean ± SD. Two‐way ANOVA with Sidak post hoc test: *P < .05; ^†^ *P* < .01; ^‡^ *P* < .001

No significant changes between any of the six training groups were observed in the concentration of the sum of nitrate and nitrite (NO_2_; Figure 6A). Concentration of NO_2_ alone was significantly higher in SupraN compared to MaxN (4.5 ± 1.9 µmol L^−1^ vs 2.9 ± 0.9 µmol L^−1^, *P* < .05; Figure 6B). No other significant differences were observed between the other groups.

Regarding oxidative stress markers, no significant changes between any of the six training groups were observed in AOPP (Figure 6C) and in XO (Figure 6D). As shown in Figures 6E, 3‐NT concentration was significantly decreased in MaxH and SupraH compared to the same intensities in normoxia (*P* < .05 and *P* < .001, respectively), and in SupraH compared to LowH (*P* < .01).

No significant differences in plasma SOD (Figure 6F) and catalase activities (Figure 6G) were observed between LowH and LowN, MaxH and MaxN, or SupraH and SupraN. SupraH mice exhibited higher plasma SOD (*P* < .05) and catalase (*P* < .01) activities than LowH (Figure 6F,G).

## 3 DISCUSSION

The main findings of this study were as follows: (a) When performed in hypoxia, independently of intensity, exercise training improved vascular reactivity (ie, both vasorelaxation and vasoconstriction) to a larger extent than the same intensity in normoxia; (b) high (ie, maximal and supramaximal) exercise training intensities performed in hypoxia improved endothelium‐dependent vasorelaxation to a greater extent than low‐intensity exercise training in hypoxia; (c) low‐intensity exercise in hypoxia induced larger endothelium‐independent relaxation than the same intensity in normoxia; (d) NO bioavailability was improved by the change in pro/antioxidant balance: this mechanism underlines the above reported benefits of high‐intensity exercise training in hypoxia.

The beneficial effects of low intensity exercise training on endothelial function are well documented.^8^, ^9^, ^24^, ^25^ High‐intensity exercise training has also been studied both in healthy and diseased humans, and data indicate a positive impact of high‐intensity exercise training compared to low‐intensity on vascular function.^12^, ^13^, ^26^, ^27^, ^28^ In animal models however, the superiority of high‐ versus low‐intensity remains debated. Although several studies showed that higher intensity exercise training improves vasorelaxation,^15^, ^16^, ^29^ others reported no benefits, or even a deleterious effect, when compared to low intensity.^17^, ^18^, ^19^, ^20^ In the present study, we showed no significant difference in aortic vasorelaxation among different exercise training intensities in normoxia in healthy mice. Our data are in line with those of Kim et al^30^ who reported that aortic endothelial function remained similar between C57BL/6J mice performing either high (6 sets of running at 85% MAS for 8 min followed by 2 min of active rest at 50% MAS) or low‐intensity (continuous running at 65% of MAS) training.

On the contrary, exercise training performed in hypoxia significantly improved vasodilation, compared to normoxia independently of exercise intensity. In addition, for the first time to date, we report a larger effect of higher intensities of exercise training in hypoxia on endothelium‐dependent relaxation.

Only few studies compared the impact of exercise training in hypoxia on endothelium‐dependent vasorelaxation. Reboul et al^31^, ^32^ reported enhanced aortic endothelium‐dependent vasorelaxation in rats that lived and trained in normoxia, but not in rats living and training in hypoxia (partial pressure of inspired O_2_ = 105 mm Hg). Authors speculated that these specific adaptations may be explained by the altitude‐induced limitation in aortic blood flow and shear stress.^31^, ^32^ This is in line with the findings of Casey and Joyner^33^ who reported in humans that the vasoconstriction activity in vascular beds of contracting muscles was blunted to a larger extent during exercise training performed in hypoxia compared to normoxia (“functional sympatholysis”), to the point where vasodilation would prevail. Moreover, post‐exercise reduction in total peripheral resistance is known for being enhanced in hypoxia (F_i_O_2_ = 0.15),^34^ suggesting a larger hypotensive effect of exercise in hypoxia (F_i_O_2_ = 0.145) than in normoxia.^35^ At the peripheral level, the main phenomenon driving blood flow response to exercise training is the vasomotor control, which translates into vasodilation and vasoconstriction.^23^

At low intensity, an important part of the observed compensatory vasodilation is mediated by vascular smooth muscle cells (VSMCs) β‐adrenergic receptors, triggering NO release. However, as exercise training intensity increases, although NO still appears to play a role in vasodilation, the β‐adrenergic‐NO pathway is taken over by other NO‐releasing mechanisms,^33^, ^36^ raising interest regarding the effects of exercise training intensity combined with hypoxia on vascular response.

During high‐intensity exercise in hypoxia, one may speculate that the compensatory vasodilation (with an increase in blood flow and shear stress), aiming at constantly maintaining total O_2_ delivery to tissues, is enhanced since exercise intensity is essential for the amplitude of this mechanism.^33^

Interestingly, the endothelium‐independent relaxation induced by the NO‐donor DEA/NO was higher at low‐intensity exercise training performed in hypoxia compared to normoxia. This indicates that the observed increased relaxation in low‐intensity exercise training combined with hypoxia may be due to an increase in soluble guanylate cyclase activity (sGC), and/or subsequent formation of the second messenger cyclic guanosine monophosphate, causing VSMCs relaxation and in turn vasodilation. However, no significant difference between our six experimental groups of mice was found with regards to aortic sGCα1 mRNA expression (Figure S2). Therefore, further investigations are needed to determine the adaptative molecular mechanisms occurring in the VSMCs in response to low intensity exercise training in hypoxia compared to normoxia.

It is important to note that the endothelium‐independent relaxation to the NO donor was not significantly different between the higher intensities exercise training in hypoxia versus normoxia (MaxH vs MaxN, and SupraH vs SupraN). The present findings suggest that the improved vasorelaxation with high intensity exercise training in hypoxia was not a consequence of a change in vasodilating capacity of VSMCs, but potentially of the production and/or bioavailability of NO.

At the molecular level, it is well known that the NO pathway is paramount in the enhancement of vasorelaxation in response to low intensity exercise, via eNOS activation and NO production.^1^, ^33^, ^36^, ^37^ Based on that, one could hypothesize that the improved ACh‐mediated vascular relaxation observed in response to exercise training in hypoxia is associated with increased eNOS expression in hypoxic compared to normoxic exercise training groups, as well as in the high‐intensity hypoxic compared to low‐intensity hypoxic exercise training groups. We therefore determined aortic mRNA and/or protein expression of eNOS and p‐eNOS. Our data revealed increased eNOS mRNA expression in hypoxic versus normoxic exercise training only with the Supra intensity. However, this result was not confirmed at the protein level. Surprisingly, we observed a lower eNOS mRNA level in MaxH versus LowH, while SupraH and LowH were not different. Again, no changes at the translational level were observed. Moreover, neither p‐eNOS protein content, nor the ratio of p‐eNOS to eNOS ratio were modulated by hypoxia and/or training intensity. In agreement with these data, circulating NO metabolism end‐products nitrate and nitrite did not reveal any significant differences among groups. Taken together, our findings indicate that the observed improved ACh‐induced relaxation may not be the result of an increased eNOS activation and/or NO production, but rather of mechanisms involved in NO bioavailability.

In order to further investigate the underlying mechanisms of this bioavailability, we looked at markers of oxidative stress and antioxidant defenses, since endothelial NO inactivation is determined by the balance between pro‐ and antioxidant status. The major source of oxidative stress in the arterial wall is NADPH oxidase (NOX), which is involved in the production of ROS, in particular of superoxide anion ($O_{2}^{\cdot ‐}$).^38^ $O_{2}^{\cdot ‐}$ reacts with NO to generate a more potent oxidant peroxynitrite (ONOO^−^).^39^ A decrease in NOX expression and/or activity associated with less $O_{2}^{\cdot ‐}$ production in the endothelium and subsequent scavenging of NO may result in increased NO availability and endothelium‐dependent vasorelaxation.

In the present study, mRNA and protein expression of p47phox—ie, a subunit of NOX2 that has been shown to be involved in pro‐oxidant activity and regulated by exercise training^40^—was not modulated by the present exercise training modalities. Besides, circulating pro‐oxidants AOPP and XO were not significantly different among groups. Very interestingly, we observed a lower nitrotyrosine (3‐NT) level with hypoxic high intensities exercise training (MaxH and SupraH) compared to the same intensities performed in normoxia, as well as in SupraH compared to LowH, indicating that oxidation of NO by ROS is decreased. This finding suggests that a limiting NO inhibition at the systemic level may be a potential mechanism for the improved endothelium NO‐dependent vasorelaxation in these mice.

SOD isoforms represent a major defense against NO inactivation and ONOO^−^ formation. SOD isoforms expression upregulation has been shown to be associated with improved endothelial function in response to exercise training in mice.^41^ Here, we did not observe any difference in aortic mRNA and/or protein expression of SOD1, SOD2 and SOD3. At the plasma level however, there was an increase in SOD and catalase activities in SupraH when compared to LowH. Based on the present 3‐NT results, one could also expect a higher SOD and catalase plasma concentration in MaxH compared to MaxN and in LowH compared to LowN. The fact that the increases in SOD and catalase activities occurred only in SupraH versus LowH suggests that this mechanism (ie, higher systemic antioxidant defense) is likely to underlie the larger improvement in endothelium‐dependent relaxation observed specifically in SupraH versus LowH.

In order to produce NO, eNOS needs the cofactor tetrahydrobiopterin (BH_4_). In its absence, eNOS will produce $O_{2}^{\cdot ‐}$ instead of NO (ie, eNOS uncoupling), impairing its bioavailability.^37^ Studies have shown a cardioprotective role of ischemic preconditioning (ie, intermittent exposure of tissues to short bouts of localized hypoxia),^42^ which is partly physiologically comparable to our SupraH training. This phenomenon would prevent an increase in $O_{2}^{\cdot ‐}$ derived from eNOS uncoupling, probably because of an increased BH_4_ availability. However, this eNOS uncoupling could be a way to limit the amount of eNOS‐dependent ROS production. By limiting the synthesis of NO, eNOS uncoupling prevents the reaction between NO and $O_{2}^{\cdot ‐}$ that in turn produces ONOO^−^,^43^ which may explain the decreased 3‐NT in SupraH and MaxH compared to the same intensities in normoxia.

Finally, we explored the possible role of hydrogen sulfide (H_2_S) in the observed vasorelaxation, since it has been previously reported that this molecule can be modulated by exercise training.^44^ Like NO, H_2_S is a ubiquitous second messenger molecule with important roles in the vessel wall, including vasoreactivity. To this end, we looked at the mRNA levels of the three enzymes responsible for H_2_S generation, ie, cystathionine β‐synthase (CBS), cystathionine γ‐lyase (CSE) and 3‐mercaptopyruvate (3MST; Figure S3). The mRNA levels of CBS, CSE and 3MST did not differ between any of the groups. Collectively, our data suggest that the H_2_S pathway is not likely involved in the improved endothelium‐dependent vasodilation observed after exercise training in hypoxia compared to the same intensities in normoxia, and higher intensities compared to the lowest intensity in hypoxia.

Another important finding of the present study is that, alongside an improved vasorelaxation, we observed an increased Phe‐induced vasoconstriction in hypoxic conditions, independently of the intensity, as well as between SupraH and LowH. Our results are partially in line with the increased sympathetic vasoconstrictor activity directed towards skeletal muscle as observed with hypoxic training.^33^ Despite this increased vasoconstriction, we propose that the degree of vasodilatation prevails over the vasoconstrictor response in our mice, in accordance with previous studies.^33^

Because Phe induces VSMCs contraction and vessels vasoconstriction by binding to α_1_‐AR on VSMCs,^45^ we hypothesized that exercise training in hypoxia would lead to increased α_1_‐AR expression. To gain insights into the molecular mechanisms, we therefore looked into aortic α_1_‐AR mRNA and protein expression. No significant differences were noted in α_1_‐AR mRNA expression between hypoxia and normoxia for the same exercise training intensities. Contrary to our expectations, α_1_‐AR mRNA expression was downregulated in MaxH and SupraH compared to LowH. However, the fact that no change was observed at the protein level indicates that an increase in α_1_‐AR expression is unlikely to explain the functional effect of hypoxic training on vasoconstriction. One might not exclude however an increased sensitivity of α_1_‐AR as a mechanism of the observed increased Phe‐induced vasoconstriction. In addition, other mechanisms, such as enhanced sympathetic activation, or increased production of endothelin‐1 might likely be involved.^46^

In conclusion, the present study provides the first experimental evidence that high‐intensity exercise training in hypoxia improves vascular reactivity. Mechanistically, it appears that benefits of high intensity exercise in hypoxia on endothelium‐dependent vasorelaxation are not mediated by an increased NO production, but rather by increased NO bioavailability secondary to an increased antioxidant status.

## 4 MATERIALS AND METHODS

### 4.1 Animals

A total of 58 8‐week‐old male C57BL/6J wild‐type mice were used in this study. Mice were purchased from Charles River Laboratories (L'arbresle, France) and housed in ventilated cages under a 12‐h light/dark cycle and in a temperature and humidity‐controlled environment. Mice had free access to a standard chow (Kliba Nafag, Switzerland) and water throughout the study.

All experiments were conducted according to Swiss animal experimentation laws and guidelines and were approved by an internal animal experimentation committee as well as the Veterinary Office of the Canton de Vaud (authorization VD3224).

### 4.2 Study design and exercise protocols

Mice were randomly divided into 6 groups: (a) low‐intensity continuous‐training in normoxia (**LowN**, n = 10); (b) low‐intensity continuous‐training in hypoxia (**LowH**, n = 9); (c) maximal‐intensity interval‐training in normoxia (**MaxN**, n = 10); (d) maximal‐intensity interval‐training in hypoxia (**MaxH**, n = 10); (e) supramaximal‐intensity repeated‐sprints training in normoxia (**SupraN**, n = 9); and (f) supramaximal‐intensity repeated‐sprints training in hypoxia (**SupraH**, n = 10).

Exercise training consisted in forced treadmill running 3 times per week for 4 weeks on a mouse treadmill (Panlab LE‐8710, Bioseb, France). Low voltage stimuli (0.2 mA) and cotton swabs were used to motivate mice to run throughout the training. Mice assigned to Low exercise training (groups 1 and 2) ran continuously for 40 min at 40% of their maximal aerobic speed (MAS). Max exercise training mice (groups 3 and 4) ran 8 bouts of 1 min at 90% of their MAS, with 1 min of passive recovery between each bout. Supra exercise training mice (groups 5 and 6) ran 4 sets of 5 × 10 s sprints at 150% of MAS, with 20 s of passive recovery between each sprint. The interset rest was of 5 min of passive recovery. The low and supra protocols were performed as previously published.^47^ For all protocols, each training session began with a 5 min warm‐up at 8 cm/s, followed by 5 min at 12 cm/s or 15 cm/s. Max and Supra mice were subjected to a cool‐down period at the end of each exercise training in order to match the total workload of the low groups. The treadmill was placed in a home‐made chamber with a fraction of inspired O_2_ (F_i_O_2_) of either 0.13 (hypoxic training) or 0.21 (normoxic training) as previously described.^47^

At the end of the 4 weeks of exercise training and 24 h after the last training session, blood was collected by cardiac puncture under isoflurane inhalation anesthesia (3%–3.5% in O_2_ for induction, 2.5% in O_2_ for maintenance; Attane Isoflurane ad us. vet., Piramal Healthcare Limited, India) and the plasma was obtained by centrifugation for 10 min at 2500 rpm at +4°C. Plasma samples were then snap frozen and stored at −80°C until further analysis. Immediately after cardiac puncture, mice were euthanized by cervical dislocation, then aortas were isolated and cleaned of fat and connective tissue in cold phosphate‐buffered saline. Tissues were either immediately used for ex‐vivo vasoreactivity studies or immersed in RNA later (Ambion, Invitrogen, California, USA) at +4°C before storage at −80°C for further real‐time reverse‐transcriptase polymerase chain reaction and western blot analyses.

### 4.3 Treadmill incremental test

Each mouse performed an incremental test to exhaustion to determine individual MAS as previously described.^47^ After a 5‐min warm‐up at 8 cm/s, the running speed was increased by 2 cm/s every 3 min until exhaustion. Mice were considered exhausted when they failed to maintain the running speed (ie, when they stayed for 2 consecutive s on the electric grid at the rear of the treadmill or when they received a total of 100 electric shocks). Mice were acclimated to treadmill running for a week before the incremental test. Mean MAS was not different between the different groups: LowN: 41 ± 6 cm/s; LowH: 38 ± 4 cm/s; MaxN: 41 ± 5 cm/s; MaxH: 43 ± 4 cm/s; SupraN: 44 ± 4 cm/s and SupraH: 44 ± 5 cm/s.

### 4.4 Vascular reactivity tension studies

Ex vivo vasoconstriction and vasodilation studies were performed using isolated vessel tension experiments, as previously described in details.^47^ The aorta was cut into vascular rings of 1.0‐2.0 mm long and mounted on two 0.1 mm‐diameter stirrups passing through the lumen. The rings were suspended in vertical organ chambers filled with 10‐mL modified Krebs‐Ringer bicarbonate (KRB) solution (118.3 mM NaCl, 4.7 mM KCl, 2.5 mM CaCl_2_, 1.2 mM MgSO_4_, 1.2 mM KH_2_PO_4_, 25.0 mM NaHCO_3_, and 11.1 mM glucose) maintained at 37°C and aerated with 95% O_2_‐5% CO_2_ (pH 7.4). Isometric tension was continuously recorded with a strain gauge system (PowerLab/8SP, ADInstruments). Afterwards, the vessel rings were progressively brought to their optimal resting tension by two steps of stretch (to 2 g)‐equilibration‐wash. Potassium chloride (KCl, 10^−1^ M) was then added to the organ chambers to test the viability of the vessels.

Vasoconstriction responses of vessel rings were assessed by addition of cumulative doses of phenylephrine (Phe; 10^−9^ to 10^−4^ M). After washout, vasorelaxation responses were then assessed, after a precontraction with Phe (10^−4^), by addition of cumulative doses of the endothelium‐dependent vasodilator acetylcholine (ACh; 10^−9^ to 10^−4^ M). Endothelium‐independent relaxation was also investigated using cumulating doses of the NO donor diethylamine DEA/NO (10^−9^ to 10^−4^ M), in the presence of the eNOS inhibitor *N* ^G^‐nitro‐L‐arginine to exclude possible interference of endogenous NO. Vasorelaxation results were expressed as percentages of the initial contraction induced by Phe. Areas under the curve (AUC) for ACh‐, DEA/NO‐ and Phe‐induced responses were calculated from the concentration‐response plots using GraphPad Prism version 6.05 (GraphPad Software, Inc, San Diego, CA, USA). All these experiments were performed in the presence of indomethacin (10^−5^ M), a cyclooxygenase activity inhibitor, to avoid possible interference of endogenous prostanoids with the vascular responses.

### 4.5 Real‐time reverse‐transcriptase polymerase chain reaction

Total RNA from aortic tissue was extracted using the RNeasy Micro Kit (Qiagen, Switzerland) according to the manufacturer's protocol. RNA was then reverse transcribed using PrimeScript RT Reagent Kit (TaKaRa Bio Inc, Japan). Quantitative real‐time polymerase chain reaction was performed on a CFX96TM real‐time system (Bio‐Rad, Switzerland) with SYBR premix Ex Taq (TaKaRa Bio Inc, Japan), according to the manufacturer's protocols. mRNA expression of the following genes was detected: eNOS, SOD3, NADPH oxidase subunit p47phox, α_1_‐AR, hypoxia inducible factor‐1 alpha (HIF‐1α), sGCα1, CBS, CSE, 3‐mercaptopyruvate sulfurtransferase (3MST), and the housekeeping gene 36B4. Specific sequences of mouse primers are shown in Table S1. For each sample, expression of target genes was normalized to the expression of the housekeeping gene 36B4 (∆Ct). Data were presented using the formula 2^−∆Ct^.

### 4.6 Western blot analyses

Frozen aortas were grinded into powder using a ceramic mortar and pestle kept in liquid nitrogen. Total proteins were then extracted using a lysis buffer containing Nonidet P‐40 0.5%, NaCl 150 mM, Na‐orthovanadate 1 mM, NaF 10 mM, Tris‐HCL pH = 7.5 10 mM, PMSF 1 mM, EDTA pH = 8.0 1 mM, aprotinin 10 µg mL^−1^, pepstatin 1 µg mL^−1^ and leupeptin 10 µg mL^−1^, followed by sonication. Protein concentration was measured using a BCA protein assay kit (Pierce BCA Protein Assay Kit, Thermo Fisher Scientific, Switzerland). 15 µg of these total proteins were separated using SDS‐PAGE, then transferred to nitrocellulose membranes (Biorad) and blocked for 1 h at room temperature using Odyssey blocking buffer mixed with TBS buffer (1:1, LI‐COR Biosciences, Bad Homburg, Germany). The membranes were probed overnight at 4°C with the following primary antibodies: mouse anti‐eNOS/NOS Type III (1:500, 140 kDa, #610297, BD Transduction Laboratories, USA), mouse anti‐eNOS pS1177 (1:500, 140 kDa, #612392, BD Transduction Laboratories, USA), rabbit anti‐superoxide dismutase 1 (SOD1; 1:1000, 18 kDa, ab13498, Abcam, UK), rabbit anti‐superoxide dismutase 2 (SOD2; 1:5000, 25 kDa, ab13533, Abcam, UK), rabbit anti‐superoxide dismutase 3 (SOD3; 1:500, 35 kDa, ab83108, Abcam, UK), goat anti‐NCF1/p47‐phox (1:500, 45 kDa, ab795, Abcam, UK), rabbit anti‐alpha 1 adrenergic receptor (α_1_‐AR; 1:500, 80 kDa, ab3462, Abcam, UK), and mouse anti‐tubulin (1:10 000, 55 kDa, Sigma Aldrich, USA). Blots were incubated with the adequate secondary antibodies for 1 h at room temperature: donkey anti‐mouse IRDye 800 (1:10 000, Rockland Immunochemicals, USA), goat anti‐rabbit Alexa 680 (1:10 000, Molecular Probes, USA) or donkey anti‐goat Alexa 594 (1:5000, Invitrogen). The immunoblot signals were detected and quantified with the Odyssey infrared imaging system (LI‐COR Biosciences, Bad Homburg, Germany). Individual values were normalized with the expression of tubulin, and then expressed as the percentage of the protein expression values obtained in the LowN group.

### 4.7 Circulating NO metabolites and pro‐ and antioxidant markers

As described previously,^48^ NO metabolism was determined as the sum of nitrite and nitrate concentrations. After reduction of nitrates by nitrate reductase, the sum of nitrate and nitrite (NOx) fluorometric quantification was based on the reaction of nitrite with 2,3‐diaminonaphtalene. The intra‐assay coefficient of variation was 5.4%. The same technique was used to measure nitrites (NO_2_), without the addition of nitrate reductase. Plasma advanced oxidation protein products (AOPP) were measured according to the semi‐automated methods developed by Witko‐Sarsat et al.^49^ Using spectrophotometry, the AOPP plasma concentrations were determined and calibrated with a chloramine‐T solution, which, given the presence of potassium iodide, absorbs at 340 nm. Absorbance was read at 340 nm and AOPP concentrations were expressed as µmol L^−1^ of chloramine equivalents. As described previously,^50^ the intra‐assay coefficient of variation was 5.4%. Xanthine oxidase (XO) activity was measured as described previously.^50^ Briefly, the absorbance of the complex (formazan blue) formed by nitroblue tetrazolium and the superoxide produced by XO in the sample was read at 560 nm every 30 s during 5 min. The slope of the formation of formazan blue overtime corresponded to XO activity. The intra‐assay coefficient of variation was 3.8%. Superoxide dismutase (SOD) activity was measured using the Oberley and Spitz method.^51^ The degree of inhibition of the reaction between superoxide radicals, produced by a hypoxanthine–xanthine oxidase system, and nitroblue tetrazolium, determined SOD activity. The intra‐assay coefficient of variation was 5.6%. The Johansson and Borg^52^ method was used to determine catalase activity, with H_2_O_2_ as a substrate, and formaldehyde as a standard. Catalase activity was determined by the rate of formaldehyde formation, induced by the reaction of methanol and H_2_O_2_. The intra‐assay coefficient of variation was 5.6%. Finally, concentrations of plasma nitrotyrosine (3‐NT), as end product of protein nitration by peroxynitrite ONOO^•−^, were measured by ELISA as previously described.^53^ The intra‐assay coefficient of variation is 6.8%.

### 4.8 Statistical analysis

All data are presented as mean ± standard deviation (SD). Data were analysed using a two‐way (O_2_ level × exercise intensity) analysis of variance (ANOVA) followed by a post‐hoc Sidak's multiple comparisons test. All statistical analyses were performed using GraphPad Prism version 6.05 (GraphPad Software, Inc, San Diego, CA, USA), and a value of *P* < .05 was considered to be statistically significant.

## 5 CLINICAL APPLICATIONS

Our findings highlight that high‐intensity exercise in hypoxia may represent a novel therapeutic strategy to improve and/or preserve endothelial function. High‐intensity training in hypoxia may bring new promising perspectives in terms of physical activity prescription amongst the general population for primary prevention of endothelial dysfunction and cardiovascular diseases, including hypertension.

## Supporting information

Fig S1

Fig S2

Fig S3

Supplementary Material

## Data Availability

The data that support the findings of this study are available from the corresponding author upon reasonable request.
