# Supplementary figures and images for: High‐intensity exercise in hypoxia improves endothelial function via increased nitric oxide bioavailability in C57BL/6 mice
Source: Acta Physiol (Oxf). 2021 Jun 19;233(2):e13700. doi: 10.1111/apha.13700 (PMC8518730; doi:10.1111/apha.13700)

Interaction = 0.1553

Exercise intensity = 0.0029

O<sub>2</sub> level = 0.0296

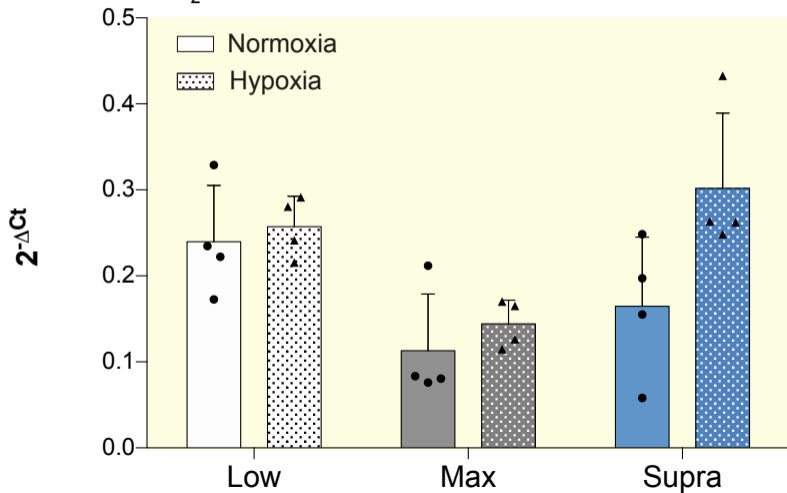

Supplement: Supplementary file 1 — Fig S1 [file APHA-233-e13700-s001.pdf]

Interaction = 0.0631

Exercise intensity = 0.5941

O<sub>2</sub> level = 0.9830

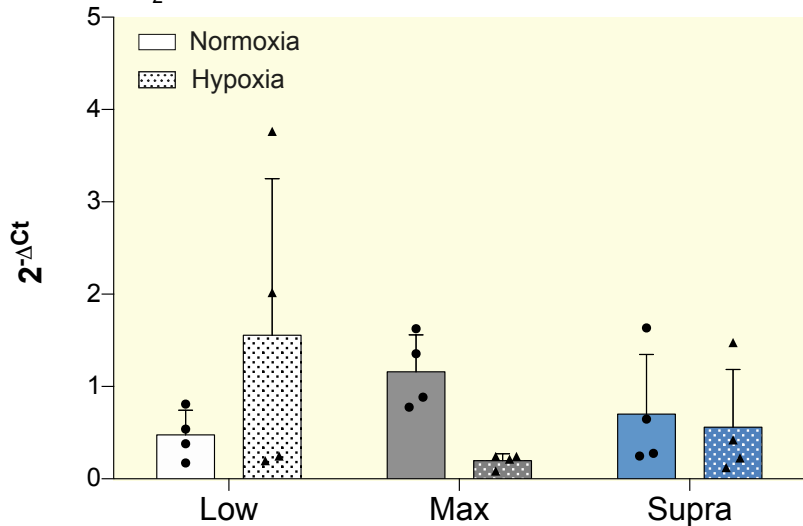

Supplement: Supplementary file 2 — Fig S2 [file APHA-233-e13700-s003.pdf]
