# Supplementary material for: High‐intensity exercise in hypoxia improves endothelial function via increased nitric oxide bioavailability in C57BL/6 mice
Source: Acta Physiol (Oxf). 2021 Jun 19;233(2):e13700. doi: 10.1111/apha.13700 (PMC8518730; doi:10.1111/apha.13700)
Supplement: Supplementary file 3 — Fig S3 [file APHA-233-e13700-s002.pdf]

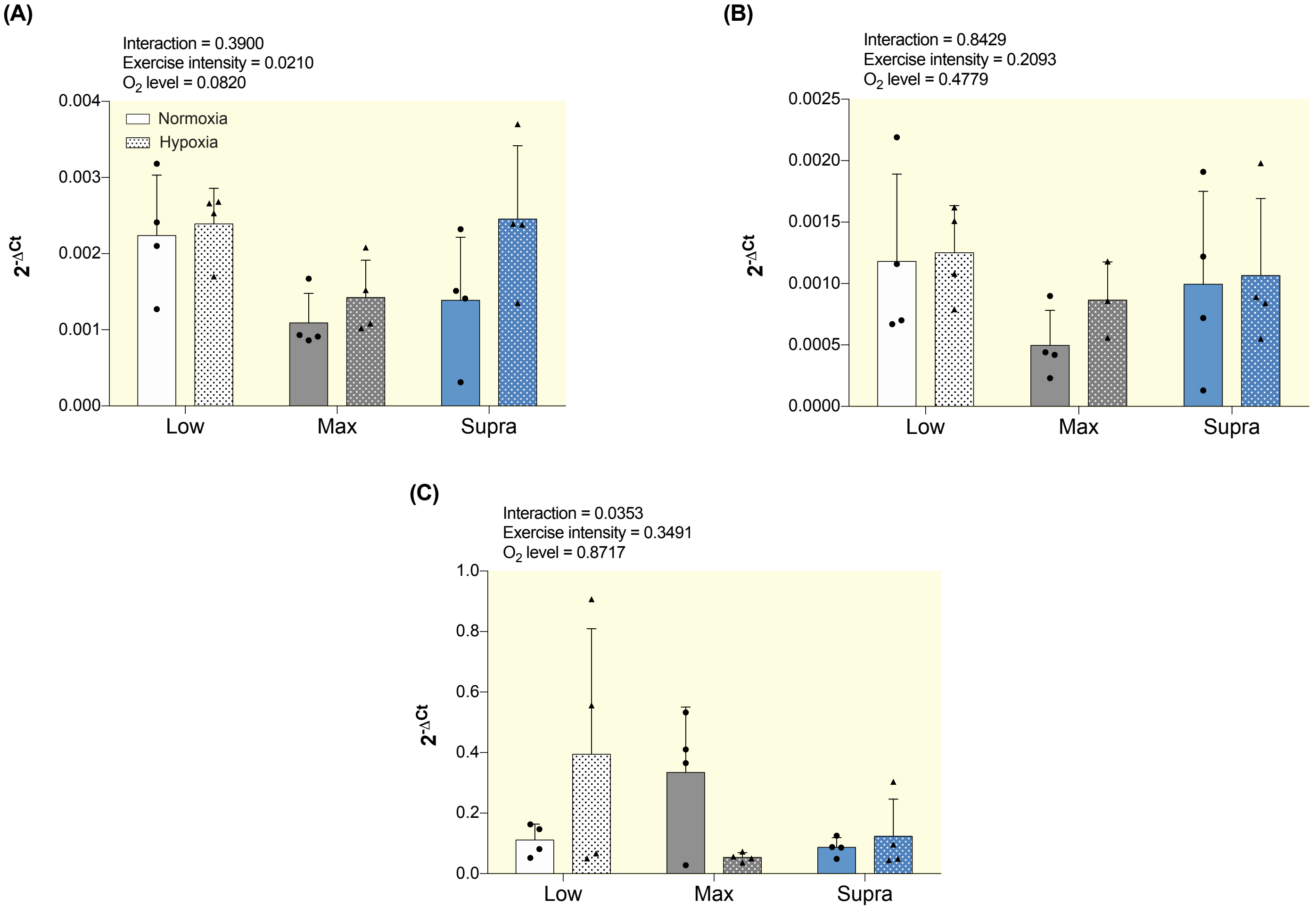
(A)
Interaction = 0.3900
Exercise intensity = 0.0210
O2 level = 0.0820
Normoxia
Hypoxia
2-ΔCt
0.000
0.001
0.002
0.003
0.004
Low
Max
Supra
(B)
Interaction = 0.8429
Exercise intensity = 0.2093
O2 level = 0.4779
2-ΔCt
0.0000
0.0005
0.0010
0.0015
0.0020
0.0025
Low
Max
Supra
(C)
Interaction = 0.0353
Exercise intensity = 0.3491
O2 level = 0.8717
2-ΔCt
0.0
0.2
0.4
0.6
0.8
1.0
Low
Max
Supra
