# Supplementary material for: High‐intensity exercise in hypoxia improves endothelial function via increased nitric oxide bioavailability in C57BL/6 mice
Source: Acta Physiol (Oxf). 2021 Jun 19;233(2):e13700. doi: 10.1111/apha.13700 (PMC8518730; doi:10.1111/apha.13700)
Supplement: Supplementary file 4 — Supplementary Material [file APHA-233-e13700-s004.docx]

**SUPPLEMENTAL DATA TABLES AND LEGENDS**

| **Gene** | **Forward sequence** | **Reverse sequence** |
| --- | --- | --- |
| 36B4 | 5’-ATGGGTACAAGCGCGTCCTG-3’ | 5’-GCCTTGACCTTTTCAGTAAG-3’ |
| eNOS | 5’-GACCCTCACCGCTACAACAT-3’ | 5’-CTGGCC TTCCGCTCATTTTC-3’ |
| SOD3 | 5’-TTCTACGGCTTGCTACTGGC-3’ | 5’-GCTAGGTCGAAGCTGGACTC-3’ |
| p47phox | 5’-AGGAGATGTTCCCCATTGAGG-3’ | 5’-CAGTCCCATGAGGCCGTTGAA-3’ |
| α1-AR | 5’-GCGGTGGACGTCTTATGCT-3’ | 5’-TCACACCAATGTATCGGTCGA-3’ |
| HIF-1α | 5’-TCAAGTCAGCAACGTGGAAG-3’ | 5’-TATCGAGGCTGTGTCGACTG-3’ |
| sGCα1 | 5’-CCCCTGGTCAGGTTCCTAAG-3’ | 5’-GGAGACTCCCTTCTGCATTCT-3’ |
| 3-MST | 5’-GGCCACCACTCTGTGTCATT-3’ | 5’-GGAGCTGATTGGCAGGTTCT-3’ |
| CBS | 5’-GGGACAAGGATCGAGTCTGGA-3’ | 5’-AGCACTGTGTGATAATGTGGG-3’ |
| CSE | 5’-TTGGATCGAAACACCCACAAA-3’ | 5’-AGCCGACTATTGAGGTCATCA-3’ |

**Supplementary Table 1.** List of primers sequences used for mRNA expression analyzes.

|  | **LowN**  (n = 10) | **LowH**  (n = 9) | **MaxN**  (n = 10) | **MaxH**  (n = 10) | **SupraN**  (n = 9) | **SupraH**  (n = 10) | ***P value*** | | |
| --- | --- | --- | --- | --- | --- | --- | --- | --- | --- |
|  |  |  |  |  |  |  | *Exercise intensity* | *O_2_ level* | *Interaction* |
| Body weight gain (% of baseline) | 11.1 ± 4.6 | 18.9 ± 3.0* | 12.8 ± 2.8 | 19.1 ± 5.1^†^ | 11.9 ± 3.1 | 15.1 ± 4.6 | *0.1773* | *< 0.0001* | *0.2097* |
| Mean blood pressure response (% of baseline) | 1.9 ± 15.0 | 3.8 ± 18.9 | 1.4 ± 12.6 | -6.5 ± 11.9 | 6.5 ± 8.3 | -2.0 ± 11.1 | *0.4091* | *0.3870* | *0.1735* |

**Supplementary Table 2. Effect of hypoxic training at different intensities on body weight and arterial blood pressure.**

Data are mean ± SD. The number of mice per group is in parentheses. Body weight gain and mean blood pressure response were expressed as percentage of change from baseline values (measured prior to the first ExT). Groups: low intensity training group in normoxia (LowN), low intensity training group in hypoxia (LowH), maximal intensity training group in normoxia (MaxN), maximal intensity training group in hypoxia (MaxH), supramaximal intensity training group in normoxia (SupraN), and supramaximal intensity training group in hypoxia (SupraH). Two-way ANOVA with Sidak post-hoc test: * p<0.001 vs LowN; ^†^ p<0.01 vs MaxN.

**Supplementary Figure 1.** mRNA expression of hypoxia inducible factor 1α in aortas of low intensity training group in normoxia (LowN), low intensity training group in hypoxia (LowH), maximal intensity training group in normoxia (MaxN), maximal intensity training group in hypoxia (MaxH), supramaximal intensity training group in normoxia (SupraN) and supramaximal intensity training group in hypoxia (SupraH). Results are expressed as 2^-ΔCt^ using 36B4 as housekeeping gene. Data are presented as mean ± SD (n = 4 mice per group). Two-way ANOVA with Sidak post-hoc test: * p<0.05, hypoxia main effect.

**Supplementary** **Figure 2.** mRNA expression of soluble guanylate cyclase subunit alpha 1 in aortas of low intensity training group in normoxia (LowN), low intensity training group in hypoxia (LowH), maximal intensity training group in normoxia (MaxN), maximal intensity training group in hypoxia (MaxH), supramaximal intensity training group in normoxia (SupraN), and supramaximal intensity training group in hypoxia (SupraH). Results are expressed as 2^-ΔCt^ using 36B4 as housekeeping gene. Data are presented as mean ± SD (n = 4 mice per group). Two-way ANOVA with Sidak post-hoc test.

**Supplementary** **Figure 3.** mRNA expression of cystathionine β-synthase (CBS, **a**), cystathionine γ-lyase (CSE, **b**) and 3-mercaptopyruvate (3MS, **c**) in aortas of low intensity training group in normoxia (LowN), low intensity training group in hypoxia (LowH), maximal intensity training group in normoxia (MaxN), maximal intensity training group in hypoxia (MaxH), supramaximal intensity training group in normoxia (SupraN), and supramaximal intensity training group in hypoxia (SupraH). Results are expressed as 2^-ΔCt^ using 36B4 as housekeeping gene. Data are presented as mean ± SD (n = 3 to 4 mice per group). *Two-way ANOVA with Sidak post-hoc test.*
